# Individual vocal recognition across taxa: a review of the literature and a look into the future

**DOI:** 10.1098/rstb.2019.0479

**Published:** 2020-05-18

**Authors:** Nora V. Carlson, E. McKenna Kelly, Iain Couzin

**Affiliations:** 1 Department of Collective Behaviour, Max Planck Institute of Animal Behavior, D-78457 Konstanz, Germany; 2 Centre for the Advanced Study of Collective Behaviour, University of Konstanz, Universitätsstraße 10, D-78457 Konstanz, Germany; 3 Department of Biology, University of Konstanz, Universitätsstraße 10, D-78457 Konstanz, Germany

**Keywords:** individual vocal recognition, recognition, bioacoustics, vocal behaviour

## Abstract

Individual vocal recognition (IVR) has been well studied in mammals and birds. These studies have primarily delved into understanding IVR in specific limited contexts (e.g. parent–offspring and mate recognition) where individuals discriminate one individual from all others. However, little research has examined IVR in more socially demanding circumstances, such as when an individual discriminates all individuals in their social or familial group apart. In this review, we describe what IVR is and suggest splitting studies of IVR into two general types based on what questions they answer (IVR-singular, and IVR-multiple). We explain how we currently test for IVR, and many of the benefits and drawbacks of different methods. We address why IVR is so prevalent in the animal kingdom, and the circumstances in which it is often found. Finally, we explain current weaknesses in IVR research including temporality, specificity, and taxonomic bias, and testing paradigms, and provide some solutions to address these weaknesses.

This article is part of the theme issue ‘Signal detection theory in recognition systems: from evolving models to experimental tests’.

## 1. Introduction

### (a) Hello and you are…?

Why do animals evolve vocal calls to distinguish individuals from one another? Many species do not exhibit individual recognition let alone individual vocal signatures, and this lack of vocal identifier does not stop them from finding mates, avoiding predators and competitors and raising offspring. In fact, vocalizing (i.e. producing sound through vibrations using the respiratory system—or swim bladders in fish—used in communication) may increase the predation risk for the producers [1]. Certain types of vocalizations (such as song) can also be cognitively demanding [2,3], requiring nutrients and time [4]. Yet, individual vocal recognition (IVR), which is the ability to recognize an individual from others owing to distinctive acoustic features, has evolved multiple times across the animal kingdom and is frequently found in mammals [5,6] and birds [7,8] and often in amphibians [9]. Producing individually distinct vocalizations is likely present in almost all vocalizing species as each individual will have slightly different vocal production apparatus owing to their unique morphology. Many studies have shown individual differences in vocalizations of mammals [5], birds [7,8], amphibians [10], fish [11,12] and reptiles [13]. However, although individuals of a species may produce distinct vocalizations, receivers may not recognize the vocalizing individual as unique [14,15]. Therefore, in this review, we focus specifically on those systems where experiments have shown some form of individual discrimination/recognition by receivers. We discuss the current state of the study of IVR, the theories behind how and why IVR evolves and areas where we need further research to understand the evolution and the use of IVR in nature.

## 2. What is individual vocal recognition?

To answer the question ‘what is individual vocal recognition and who performs it’, we need to first make the distinction between recognition and discrimination. Recognition is a mental process whereby individuals can tell apart other individuals based on some distinctive cue [8]. As this is a mental process, it cannot be measured (though measuring brain activity may provide some insights). However, we can measure an individual responding differently (behaviourally or physiologically) to different individuals, i.e. discrimination [8].

**Figure 1.**
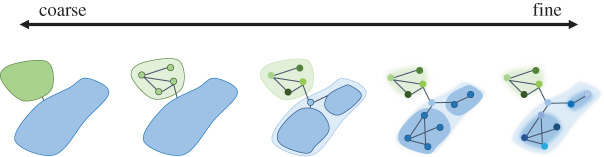
Levels of recognition from coarse to fine that are found among the animal kingdom. Moving along the scale from coarse to fine involves being able to recognize finer and finer categories of groups or individuals. Illustrated as networks of relationships. (Online version in colour.)

We also need to define what we mean by individual recognition. Recognition systems in general exist along a spectrum where an individual may be able to recognize coarse or fine scale groups (coarse: juvenile versus adult, male versus female; fine: member of flock versus stranger, related versus unrelated) or specific individuals (i.e. different offspring, mates, social partners; figure 1) [16–18]. Individual recognition (IR), a type of the recognition system, involves one individual responding to another as a unique entity owing to its distinctive characteristics [17,19,20]. In general, there are two groups of thought regarding what constitutes IR. The first is *IR-singular* (box 1). In this context, as long as the recognizer can tell one individual apart from others, this constitutes individual recognition [15,17]. IR-singular is very common in neighbour–stranger contexts, where an individual neighbour is recognized over strangers (i.e. North American bullfrog, *Rana catesbeiana* [21]), or in mate recognition situations, where the mate is recognized over strangers (i.e. laughing gulls, *Larus atricilla* [7]). The second is *IR-multiple* (box 1). In this context, receivers must be able to recognize multiple individuals (i.e. each individual in a group) apart from one another in order to be considered to have performed IR [20]. IR-multiple may be more common in highly social species with a strong dominance hierarchy or where repeated interactions with individuals are common, resulting in a benefit for receivers who are able to discriminate between multiple individuals. We see this type of recognition tested in social mammals (e.g. Japanese monkeys, *Macaca fuscata* [22] and giant otters, *Pteronura brasiliensis* [23]) and social birds (e.g. European starlings, *Sturnus vulgaris* [24] and noisy miners, *Manorina melanocephala* [25]), often with some types of acoustic discrimination tasks, rather than natural behaviour.

Box 1. **Glossary of Terms.**

**IR:** Individual recognition occurs when an individual can recognize another individual based on unique characteristics.

**IVR**: Individual vocal recognition occurs when an individual can recognize another individual's vocalization (or stridulation in the case of many fish and insects) based on unique characteristics of their vocalization.

**IR/IVR-singular:** IVR in which an individual recognizes one other individual from all others.

**IR/IVR-multiple:** IVR in which an individual recognizes multiple individuals, often within a social group.

## 3. How do we test for individual vocal recognition?

When testing for IVR using behavioural responses from individuals (i.e. not only examining differences in vocalizations), the majority of experiments use some form of playback design. Playbacks ensure that individuals are responding to the vocalizations (i.e. acoustic cues) rather than other cues (i.e. visual, chemical, etc.) as many species use multiple cues for individual recognition [17,26]. In addition, although individual recognition can involve multiple cues [18], vocal cues alone should allow for individual recognition owing to their ability to travel distances and through barriers that may impede the use of other cues (i.e. low-light conditions, physical separation, visual separation; e.g. howler monkeys, *Alouatta pigra*, in a forest [27] and African elephants, *Loxodonta africana*, across many kilometres [28]).

There are three major approaches for testing of IVR using playbacks (box 2), each with their strengths and weaknesses. One is the natural playback approach, trying to elicit natural response behaviour to the vocalizations of different individuals [34]. Another is based more on a behavioural psychology approach (box 2), using training or discrimination paradigms to directly ask an individual if a stimulus is different [22,24]. The third, habituation/dishabituation (box 2), is a combination of the previous two and uses an animal's tendency to habituate to a similar signal to then ask if the signal that we classify as different is also perceived as different by the subject [31,35].

Box 2. **Testing individual vocal recognition.**

**Natural playbacks:** playing a series of different sounds/calls to an individual or group to test their response to the acoustic stimulus and/or induce specific behaviour.

**Behavioural Psycology**: Using trained behavioural responses to stimuli known to be perceived as different (e.g. signaller sex) in order to determine whether an individual differentiates between other stimuli that share this same difference. This is often achieved through either a *Go no‐go experiment* or *Discrimination task* (see below).

*Go no-go experiment.* These experiments test whether an individual can discriminate between two categories of stimuli based on whether they respond (go) to a correct stimulus or don't respond (no-go) to an incorrect stimulus. First subjects go through a training phase with training stimuli that the experimenter knows are perceived as different. During the training phase, the subject is exposed to one of two stimuli categories to train them to respond to the difference the experimenter is looking for. If they respond correctly (i.e. tap the screen, go to a specific perch, etc.), they are rewarded (usually food). If they respond incorrectly then they are usually punished (e.g. lights go out, loud noise plays briefly, etc.). To start a new trial, the birds must respond correctly (i.e. go to a correct stimulus or no-go to an incorrect stimulus) and then wait a specific time (usually a few seconds) for a new trial to start. After the subjects reach a certain proficiency with the training stimuli (i.e. 90% correct), then they are tested using the same paradigm with new test stimuli [22,29].

*Discrimination task:* These experiments test whether an individual can discriminate between two or more categories of stimuli by choosing the representation of that stimuli (e.g. button, image, etc.). First subjects go through a shaping phase where they are trained to use the apparatus usually by getting a food reward. This is done in many different ways. In many bird species, for example, shaping will involve getting a bird to peck a button or screen using flashing lights or images of food, etc., then ramping down the stimulus that induces the pecking behaviour until the subject will peck to get a reward with no stimulus. Once the subjects readily perform the behaviour necessary to get rewarded, then discrimination training can begin. Like in go no-go experiments, the individuals are trained to discriminate between specific known categories of stimuli with training data by rewarding the individual for choosing the correct button in response to a stimulus and punishing an incorrect response. Once the subjects reach a certain proficiency with the training stimuli, then they are tested using the same paradigm with the new test stimuli [24,30].

**Habituation/dishabituation:** These experiments use the natural habituation response to tell if an individual can discriminate between two categories of stimuli. In these experiments, individuals will be presented with a stimuli or a set of categorically similar stimuli until they stop responding to the stimuli (i.e. habituate). Once they stop responding, then they will be presented with a new stimulus that is thought to be from a different category from the previous stimuli. If they respond to the new stimuli, then they are not habituated (i.e. dishabituated) to that stimuli and perceive it as different from the previous stimuli they have habituated to, and if they do not respond, then they are habituated to that stimulus and do not perceive it as different from the other stimuli they are habituated to [25,31–33].

The *natural* *playback approach* is often straightforward to implement and is regularly used to test specific categorical relationships (i.e. neighbour–stranger, parent–offspring, familiar–nonfamiliar and mate recognition—IR-singular; table 1). This approach is particularly useful in behavioural ecology as it focuses on naturally occurring behavioural responses to individuals, making the results ecologically relevant in the circumstances in which they were tested. For example, Petrinovich [91] used playbacks in the wild to show that Northern elephant seal, *Mirounga angustirostris*, mothers could recognize the distress calls of their pups during the peak of breeding season in their natural environment [91]. Although this approach works well in many circumstances, it does not work well when there are no specific predictable behavioural responses to the call type being used or when there are subtle variations in discrimination of similar individuals (e.g. very similar responses to different individuals of similar dominance). For example, using playbacks of contact calls, Arnold & Wilkinson [107] found that pallid bats, *Antrozous pallidus*, responded more to familiar than unfamiliar calls. This playback approach was very successful at examining the response of individuals to familiar and unfamiliar individuals but could not (and did not try to) separate out whether individuals responded differently to specific familiar individuals [107].

**Table 1.** Summary of experiments showing individual vocal recognition. Table includes experimental approach (test type), directionality of the signal (directionality), context of the IVR (type of ID), the type of IVR exhibited and the behavioural response of individuals (response). ^a^Referred to with previous classification *Cercopithecus aethiops* in the text.

taxa	common name	species name	call type	test type	directionality	type of ID	type of IVR	response	citation
amphibians	agile frogs	*Rana dalmatina*	call	playback	bi-directional	neighbour–stranger	singular	individuals called more in response to strangers’ calls than neighbours' calls	[36]
amphibians	concave-eared torrent frogs	*Odorrana tormota*	call	playback	bi-directional	neighbour–stranger	singular	individuals called in response to strangers' calls but not neighbours' calls	[9]
amphibians	dendrobatid frogs	*Colostethus beebei*	call	playback	bi-directional	neighbour–stranger	singular	males responded more aggressively to strangers' calls than neighbours' calls	[37]
amphibians	North American bullfrogs	*Rana catesbeiana*	calls	playback (synthetic calls)	bi-directional	neighbour–stranger	singular	individuals responded more aggressively to strangers' calls than neighbours' calls, regardless of where the neighbour is calling form	[21]
amphibians	North American bullfrogs	*Rana catesbeiana*	territorial call	playback	bi-directional	neighbour–stranger	singular	individuals responded more aggressively to strangers' calls than neighbours' calls	[38]
amphibians	olive frogs	*Babina adenopleura*	advertisement calls	playback	bi-directional	neighbour–stranger	singular	males responded more to strangers' calls than neighbours' calls	[39]
birds	kittiwake gulls	*Rissa tridactyla*	kittywake call	playback	bi-directional	mate	singular	individuals responded more to their mate's call than either neighbour or stranger calls	[40]
birds	laughing gulls	*Larus atricilla*	long calls and Ke-hah calls	playback	bi-directional	mate	singular	individuals responded to their mate's call but not to neighbour or stranger calls	[41]
birds	magellanic penguins	*Spheniscus magellanicus*	ecstatic and display calls	playback	bi-directional	mate	singular	females responded more to their partner's ecstatic calls than neighbours' or strangers'; pairs responded more to their partner's mutual display calls than neighbour or stranger calls	[42]
birds	silvereyes	*Zosterops lateralis*	variable, linear and short contact calls	playback	bi-directional	mate	singular	individuals responded more to their mate's call than either neighbour or stranger calls	[43]
birds	green-rumped parrotlets	*Forpus passerinus*	contact calls	playback	untested	mate	singular	females responded more to mates than others (when on nest)	[44]
birds	alder flycatchers	*Empidonax alnorum*	song	playback	bi-directional	neighbour–stranger	singular	individuals responded more aggressively to strangers' calls than neighbours' calls	[45]
birds	carolina wrens	*Thryothorus ludovicianus*	song	playback	bi-directional	neighbour–stranger	singular	individuals responded more aggressively to strangers' calls than neighbours' calls	[46]
birds	corncrakes	*Crex crex*	call	playback	bi-directional	neighbour–stranger	singular	males responded more aggressively to strangers' calls than neighbours' calls	[47]
birds	Mexican jays	*Aphelocoma ultramarina*	primary calls	playback	bi-directional	neighbour–stranger	singular	individuals responded more aggressively (more calls, shorter latency to approach) to stranger and group member calls	[48]
birds	New Zealand bellbirds	*Anthornis melanura*	song	playback	bi-directional	neighbour–stranger	singular	individuals responded more aggressively to neighbours' calls than strangers' calls	[49]
birds	ovenbirds	*Seiurus aurocapilla*	song	playback	bi-directional	neighbour–stranger	singular	males responded more aggressively to stranger males' calls than neighbour males' calls	[50]
birds	pukekos	*Porphyrio porphyrio melanotus*	crowing calls	playback	bi-directional	neighbour–stranger	singular	all (especially males) responded most to stranger males, then neighbour males, then group males	[51]
birds	red-winged fairy-wrens	*Malurus elegans*	song	playback	bi-directional	neighbour–stranger	other	females responded more strongly to strangers' calls than familiar calls	[52]
birds	skylarks	*Alauda arvensis*	song	playback	bi-directional	neighbour–stranger	singular	individuals responded more aggressively to strangers' calls than neighbours' calls, but only during the breeding season	[53]
birds	song sparrows	*Melospiza melodia*	song	playback	bi-directional	neighbour–stranger	singular	individuals responded more aggressively to strangers' calls than neighbours' calls, only when the calls were coming from the correct location	[54]
birds	stripe-backed wrens	*Campylorhynchus nuchalis*	duets	playback	bi-directional	neighbour–stranger	singular	principal pair responded more aggressively to strangers' calls than neighbours' calls and to neighbours' calls from wrong location	[55]
birds	tawny owls	*Strix aluco*	hooting	playback	bi-directional	neighbour–stranger	singular	individuals responded more to strangers' calls than neighbours' calls	[56]
birds	water rails	*Rallus aquaticus*	terrotrial calls	playback	bi-directional	neighbour–stranger	singular	individuals responded more aggressively to strangers' calls than neighbours' calls	[57]
birds	white-throated sparrows	*Zonotrichia albicollis*	song	playback	bi-directional	neighbour–stranger	singular	males responded more aggressively to strangers' calls than neighbours' calls	[58]
birds	white-throated sparrows	*Zonotrichia albicollis*	song	playback	bi-directional	neighbour–stranger	singular	individuals responded more aggressively to strangers' calls than neighbours' calls from an expected location, and responded more aggressively to neighbours' calls from an unexpected location	[59]
birds	winter wrens	*Troglodytes troglodytes*	song	playback	bi-directional	neighbour–stranger	singular	individuals responded more aggressively to neighbours' calls than strangers' calls, but only during the breeding season	[60]
birds	yellow throats	*Geothlypis trichas*	song	playback	bi-directional	neighbour–stranger	singular	males responded more aggressively to stranger males' calls than neighbour males' calls	[61]
birds	razorbills	*Alca torda*	begging and calls	playback	bi-directional	parent–offspring	singular	males responded more to own chicks' calls than stranger chicks' calls	[62]
birds	razorbills	*Alca torda*	begging and calls	playback	bi-directional	parent–offspring	singular	chicks responded more to their father's calls than to other males' calls	[62]
birds	razorbills	*Alca torda*	begging and calls	playback	bi-directional	parent–offspring	singular	females did not respond differently to their chicks' or stranger chicks' calls	[62]
birds	thick-billed murres	*Uria lomvia*	calls	playback (simultaneous presentation)	bi-directional	parent–offspring	singular	individuals responded more to their own parent/offspring's calls than neighbour or stranger's calls	[63]
birds	barn swallows	*Hirundo rustica*	calls	playback (simultaneous presentation)	uni-directional (young -> parents)	parent–offspring	singular	chicks responded more to parent calls than non-parent calls, parents didn't differentiate between their own and stranger chick calls	[64]
birds	tree swallows	*Tachycineta bicolor*	calls	playback	uni-directional (young -> parents)	parent–offspring	singular	chicks responded more to parent calls than non-parent calls, parents didn't differentiate between their own and stranger chicks' calls	[65]
birds	bank swallows	*Riparia riparia*	calls	playback (simultaneous presentation)	untested (parents -> young)	parent–offspring	singular	parents responded more to their own chicks' calls than stranger chicks' calls	[66]
birds	black redstarts	*Phoenicurus ochruros*	begging	playback	untested (parents -> young)	parent–offspring	singular	parents responded more to calls of chicks they preferentially fed compared to calls from chicks they did not preferentially feed	[67]
birds	cliff swallows	*Hirundo pyrrhonota*	begging	playback	untested (parents -> young)	parent–offspring	singular	parents preferentially respond to (visit with food) their own chicks' calls relative to stranger chicks' calls	[68]
birds	European bee eaters	*Merops apiaster*	begging	playback (simultaneous presentation)	untested (parents -> young)	parent–offspring	singular	parents and helpers preferentially approach their own chicks' calls over stranger chicks' calls	[69]
birds	bank swallows	*Riparia riparia*	calls	playback	untested (young -> parents)	parent–offspring	singular	chicks responded more to parents' calls than stranger adults' calls	[70]
birds	black-billed gulls	*Larus bulleri*	mew calls	playback	untested (young -> parents)	parent–offspring	singular	chicks responded more to parents' calls than stranger adults' calls	[34]
birds	cliff swallows	*Hirundo pyrrhonota*	calls	playback	untested (young -> parents)	parent–offspring	singular	chicks responded more to parents' calls than stranger adults' calls	[68]
birds	laughing gulls	*Larus atricilla*	multiple calls	playback	untested (young -> parents)	parent–offspring	singular	chicks responded to parents' calls but not strangers' calls	[41]
birds	laughing gulls	*Larus atricilla*	calls	playback	untested (young -> parents)	parent–offspring	singular	chicks responded to parents' calls but not to strangers' calls	[71]
birds	macaroni penguins	*Eudyptes chrysolophus*	calls	playback	untested (young -> parents)	parent–offspring	singular	chicks responded more to parents' calls than stranger adults' calls	[72]
birds	magellanic penguins	*Spheniscus magellanicus*	display calls	playback	untested (young -> parents)	parent–offspring	singular	chicks responded more to parents' calls than stranger adults' calls	[42]
birds	European starlings	*Sturnus vulgaris*	song	discrimination task	bi-directional	social	multiple	individuals discriminated between different individuals	[24]
birds	jungle crows	*Corvus macrorhynchos*	ka calls	discrimination task	bi-directional	social	multiple	individuals discriminated between different individuals	[30]
birds	noisy miners	*Manorina melanocephala*	chirr recruitment calls	habituation/dishabituation	bi-directional	social	multiple	individuals could discriminate between calls of familiar birds as well as between calls of unfamiliar birds	[25]
birds	peafowls	*Pavo cristatus*	alarm calls	habituation/dishabituation	bi-directional	social	multiple	females discriminated between different individuals	[31]
birds	great tits	*Parus major*	song	playback	untested	social	multiple	females were more likely to intrude onto a neighbour males' territory if he won a fight (playback) with her mate	[73]
birds	great tits	*Parus major*	song	go/no-go	untested	social	multiple	females discriminated between different adult males	[29]
birds	jackdaws	*Corvus monedula*	mobbing call	playback	bi-directional	social (anti-predator)	multiple	individuals responded to playbacks with larger number of callers appropriately (can differentiate callers from one another to estimate number of callers)	[74]
birds	Western Australian magpies	*Cracticus tibicen dorsalis*	alarm calls	playback	bi-directional	social (anti-predator)	multiple	individuals paid more attention to calls from reliable individuals than calls from individuals made ‘unreliable’	[75]
birds	ravens	*Corvus corax*	haa food recruitment calls	playback	bi-directional	social (foraging)	other	individuals responded to feeding calls of familiar females more than familiar males or unknown individuals	[76]
birds	long-tailed tits	*Aegithalos caudatus*	contact call	playback	bi-directional	social (kin)	other	pairs were more aggressive to calls of non-kin compared to kin	[77]
birds	spectacled parrotlets	*Forpus conspicillatus*	contact calls	playback	bi-directional	social (mate and kin)	singular	individuals respond to mates preferentially, then to siblings	[78]
birds	brown-throated conures	*Aratinga pertinax*	contact and overflying calls	playback	bi-directional	social (roosting)	other	individuals respond differently to social partners (most to mates, then to roost, then to strangers)	[79]
fish	bicolour damselfishes	*Pomacentrus partitus*	chirps	playback	bi-directional	neighbour–stranger	singular	individuals responded more aggressively to non-nearest neighbour calls than further neighbours’/strangers' calls and neighbour calls from an unexpected location	[80]
mammals	Australian fur seals	*Arctocephalus pusillus doriferus*	territorial bark call	playback	bi-directional	neighbour–stranger	singular	males responded more aggressively to stranger males' calls than neighbour males' calls	[81]
mammals	black howler monkeys	*Alouatta pigra*	howling	playback	bi-directional	neighbour–stranger	singular	individuals responded more to neighbour calls coming from an unexpected location compared to an expected location	[27]
mammals	de brazza monkeys	*Cercopithecus neglectus*	contact calls	playback	bi-directional	neighbour–stranger	singular	individuals responded more to unfamiliar individuals than familiar ones	[82]
mammals	pigmy marmosets	*Cebuella pygmaea*	contact calls	playback	bi-directional	neighbour–stranger	singular	individuals responded to familiar calls from expected locations but didn't respond to familiar calls from unexpected locations	[83]
mammals	red squirrels	*Tamiasciurus hudsonicus*	rattle calls	playback	bi-directional	neighbour–stranger	singular	individuals responded more to strangers' calls than neighbours' calls	[84]
mammals	Richardson's ground squirrels	*Spermophilus richardsonii*	alarm calls	habituation/dishabituation	bi-directional	neighbour–stranger	singular	individuals differentiated between a familiar (neighbour) and strangers' alarm calls	[33]
mammals	spotted hyenas	*Crocuta crocuta*	long-distance whoop calls	playback	bi-directional	neighbour–stranger	singular	individuals responded to playbacks with larger numbers of unfamiliar callers appropriately (can differentiate callers from one another to estimate number of callers)	[85]
mammals	domestic sheep	*Ovis aries*	calls	playback	bi-directional	parent–offspring	singular	individuals responded more to their own parent/offspring's calls than neighbour or stranger's calls	[86]
mammals	reindeers	*Rangifer tarandus*	calls	playback	bi-directional	parent–offspring	singular	mothers responded more to their own calf's calls than other calves' calls	[87]
mammals	reindeers	*Rangifer tarandus*	calls	playback	bi-directional	parent–offspring	singular	calves (when separated from mother) responded more to their mother's calls than those of a stranger mother	[87]
mammals	Mexican free-tailed bats	*Tadarida brasiliensis mexicana*	pup isolation calls and adult echolocation calls	playback	uni-directional (parents -> young)	parent–offspring	singular	mothers respond more to their own pup's calls than stranger pup's calls, pups respond to all adults the same	[88]
mammals	racoons	*Procyon lotor*	mother chitter calls, cub whistles	playback	uni-directional (young -> parents)	parent–offspring	singular	pups responded faster and called more in response to their mother's calls than stranger females' calls, mothers didn't differentiate between their own and stranger pup calls	[6]
mammals	Japanese macaques	*Macaca fuscata*	coo calls	playback	untested (parents -> young)	parent–offspring	singular	mothers responded more to calls from their own young than to calls from un-related young	[89]
mammals	little brown bats	*Myotis lucifugus*	icalls	playback (simultaneous presentation)	untested (parents -> young)	parent–offspring	singular	mothers responded more to calls of their own young than those calls of stranger young	[90]
mammals	Northern elephant seals	*Mirounga angustirostris*	distress calls	playback	untested (parents -> young)	parent–offspring	singular	mothers respond more to their own pup's calls than stranger pups' calls	[91]
mammals	spotted hyenas	*Crocuta crocuta*	long-distance whoop calls	playback	untested (parents -> young)	parent–offspring	singular	mothers responded more to their own cubs' calls than stranger cubs' calls, relatives of calling cubs were more likely to respond to calls than non-relatives	[92]
mammals	squirrel monkeys	*Saimiri sciureus*	calls	actual separated infants vocalizing	untested (parents -> young)	parent–offspring	singular	mothers responded more to their own infants' calls than to stranger infants' calls	[93]
mammals	vervet monkeys	*Cercopithecus aethiops*^a^	screams	playback	untested (parents -> young)	parent–offspring	singular	mothers respond more to their own juvenile's calls than unknown/other juveniles' calls, others present looked at the mother of the playback caller	[94]
mammals	Australian sea lions	*Neophoca cinerea*	pup attraction calls	playback	untested (young -> parents)	parent–offspring	singular	pups responded more to mother's calls than stranger females' calls	[95]
mammals	African elephants	*Loxodonta africana*	contact calls	playback	bi-directional	social	other	females could discriminate calls from group members from calls of strangers, and calls of familiar groups from calls of strangers	[96]
mammals	bottlenose dolphins	*Tursiops truncatus*	signature whistles	playback	bi-directional	social	multiple	individuals respond to playback of signature whistle without vocal characteristics (i.e. could discriminate between individuals)	[97]
mammals	chama baboons	*Papio hamadryas ursinus*	conflict vocalizations	playback	bi-directional	social	multiple	individuals paid more attention to playbacks of third-party reversed conflicts (where a dominant individual was being subordinate) than normal conflicts	[98]
mammals	Eastern chipmunks	*Tamias striatus*	chucking alarm calls	playback	bi-directional	social	other	individuals responded with increased vigilance to bolder individuals' calls	[99]
mammals	giant otters	*Pteronura brasiliensis*	contact and hums	habituation/dishabituation	bi-directional	social	multiple	individuals discriminated between different individuals	[23]
mammals	Japanese monkeys	*Macaca fuscata*	coo call	go/no-go	bi-directional	social	multiple	individuals discriminated between different individuals	[22]
mammals	rhesus macaques	*Macaca mulatta*	contact calls	habituation/dishabituation	bi-directional	social	multiple	females discriminated between different individuals	[32]
mammals	rhesus macaques	*Macaca mulatta*	calls	playback and simultaneous presentation	bi-directional	social	multiple	looked longer at the individual whose voice had been played back	[100]
mammals	vervet monkeys	*Cercopithecus aethiops*^a^	foreign troop and alarm calls	playback	bi-directional	social	multiple	individuals ignored calls of an individual who was made ‘unreliable'	[101]
mammals	yellow-bellied marmots	*Marmota flaviventris*	alarm calls	habituation/dishabituation	uni-directional (all -> adult females)	social	multiple	females discriminated between different adult females	[102]
mammals	dwarf mongooses	*Helogale parvula*	alarm calls	playback	bi-directional	social (anti-predator)	multiple	individuals responded more to sentinels than foragers	[103]
mammals	Richardson's ground squirrels	*Spermophilus richardsonii*	alarm calls	playback	bi-directional	social (anti-predator)	multiple	juveniles responded more vigilantly to callers made reliable compared to callers made unreliable	[35]
mammals	bottlenose dolphins	*Tursiops truncatus*	signature whistles	playback	bi-directional	social (familiar)	other	captive dolphins responded more to familiar whistles than unfamiliar ones	[97]
mammals	chimpanzees	*Pan troglodytes*	arrival pant hoots	playback	bi-directional	social (foraging)	multiple	males were more likely to respond to individuals with whom they had higher levels of friendship and who were of higher rank	[104]
mammals	dwarf mongooses	*Helogale parvula*	contact calls	playback	bi-directional	social (foraging)	multiple	individuals responded differently to the simulated approach of different individuals to their food source based on the approaching individual's relative dominance	[105]
mammals	meerkats	*Suricata suricatta*	close calls	playback (simultaneous presentation)	bi-directional	social (foraging)	multiple	individuals responded differently to an improbable situation (one individual calling from two locations) than a possible one (two individuals calling from two locations)	[106]
mammals	African elephants	*Loxodonta africana*	long-distance contact calls	playback	bi-directional	social (kin)	other	individuals responded more to family member calls than non-family member calls	[28]
mammals	rhesus macaques	*Macaca mulatta*	contact calls	playback and habituation/dishabituation	bi-directional	social (kin)	multiple	females responded more to familiar kin than familiar non-kin	[32]
mammals	pallid bats	*Antrozous pallidus*	contact calls	playback	bi-directional	social (roosting)	other	flying individuals respond more to calls from familiar than unfamiliar individuals	[107]
mammals	tamarins	*Saguinus mystax*	long calls	actual separated individual vocalizing	bi-directional	social (separation)	other	after separated caller called, own troop called in response more than non-troop	[108]
mammals	domestic dogs	*Canis familiaris*	human voices	playback	untested (focal species -> other species)	social (cross-species)	singular	responded more to mismatched voice–face combinations than correctly paired ones	[109]
mammals	domestic dogs	*Canis familiaris*	human voices	habituation/dishabituation	untested (focal species -> other species)	social (cross-species)	multiple	differentiated between unfamiliar individuals, even those of the same sex	[110]
mammals	domestic horses	*Equus ferus*	human voice	simultaneous presentation and playback	untested (focal species -> other species)	social (cross-species)	singular	responded more to correct voice–face combinations when person was familiar	[111]
mammals	Campbell's monkeys	*Cercopithecus campbelli*	contact calls	playback	untested (focal species -> other species)	social (cross-species)	singular	individuals responded more to unfamiliar individuals than familiar ones	[82]
mammals	cheetahs	*Acinonyx jubatus*	human voice	playback	untested (focal species -> other species)	social (cross-species)	singular	responded more to familiar than unfamiliar voices	[112]
mammals	guereza Colobus monkeys	*Colobus guereza*	contact calls	playback	untested (focal species -> other species)	social (cross-species)	singular	individuals responded more to unfamiliar individuals than familiar ones	[82]
mammals	red-capped mangabeys	*Cercocebus torquatus*	contact calls	playback	untested (focal species -> other species)	social (cross-species)	singular	individuals responded more to unfamiliar individuals than familiar ones	[82]
mammals	rhesus macaques	*Macaca mulatta*	human voice	playback and simultaneous presentation	untested (focal species -> other species)	social (cross-species)	multiple	looked longer at the individual whose voice had been played back	[100]

Although playbacks are widely used, especially in natural habitats, there are a number of drawbacks to this methodology. For some species, IVR cannot be tested using playbacks owing to logistical and technical difficulties. For other species, playbacks are too disruptive. In birds, where the use of playbacks is common, responding to recordings takes time away from foraging, requires energy and makes individuals more vulnerable to extra-pair copulations and predation [113]. Playbacks can also lead to elevations in corticosterone and testosterone levels [114,115], which may negatively affect the current breeding attempts [116]. Playbacks can alter species' behaviours, leading to increased vocalizations after the playback [117]. Therefore, in critically endangered species, the use of playbacks is discouraged so as not to disrupt normal behaviours and breeding attempts. For circumstances where playbacks are logistically impossible or will prove too disruptive, we recommend the two methods discussed in this study.

The *behavioural psychology approach* asks the receiver directly whether they can differentiate between two individuals. The focus with which the questions can be asked removes most of the doubt surrounding whether a receiver can tell specific individuals apart, and this is a very effective approach for circumstances with no specific predictable behavioural response, or with subtle variations in discrimination of similar individuals (i.e. IVR-multiple). For example, by using a go no-go experimental approach, Ceugniet & Izumi [22] found that Japanese monkeys could differentiate between the calls of different individuals [22]. Owing to the specificity with which questions can be asked using this method, the behavioural psychology approach may also be useful in asking the underlying questions about how recognition works in different species (i.e. how many individuals can a receiver recognize, what is the distinctive feature used for recognition, can receivers recognize/discriminate between unknown individuals, etc.). Although powerful, these approaches are harder to use, as many of them (i.e. discrimination tasks, go no-go, etc.) require captivity and/or shaping and training for the experiments to work (box 2). Therefore, these experimental approaches are often not as closely tied to the natural behaviour of the animal. These approaches ask if an animal *can* respond differently to individuals rather than determining if they *do* respond differently (as in the playback approach).

The *habituation/dishabituation approach* combines parts of both the playback and behavioural psychology approaches. This approach is easier to conduct in the wild than the behavioural psychology approach as the focal individual does not need to be trained or interact with any equipment. Habituation/dishabituation also allows researchers to ask more targeted questions than playbacks alone by using an individual's habituation response to see if something the experimenter considers to be a different category is perceived as different by the focal individual. By using a habituation/dishabituation approach, McDonald [25] found that noisy miners could differentiate not only between familiar individuals but also between unfamiliar ones [25]. Nichols & Yorzinski [31] also used a habituation/dishabituation experiment with alarm calls to show that peahens, *Pavo cristatus*, can tell the difference between different individuals' calls [31]. However, as with the behavioural psychology approach, experimenters need to be careful when designing habituation/dishabituation experiments to ensure that they are actually testing for recognition and differentiation between target individuals by the receiver [31,118].

## 4. Why is individual vocal recognition so widespread?

### (a) Let me introduce myself, ‘tweet, hoot, shriek!’

Individual recognition can, in principle, benefit both the signaller and the receiver, although most research to date has focused on benefits to receivers. For the vocalizer, being correctly identified by a receiver is expected to be generally beneficial when it leads to increased cooperation from kin (i.e. unique chick calls allow their parents to find and feed them; cliff swallows, *Hirundo pyrrhonota* [64]; tree swallows, *Tachycineta bicolor* [65]; Australian sea lion, *Neophoca cinerea* [67]) or reinforces a reciprocal relationship with a receiver or cooperation within a group (i.e. performing food calls to alert approaching members of the group; chimpanzees, *Pan troglodytes* [104]). Being correctly identified by a receiver can also reduce aggression towards the signaller during territorial (‘dear enemy’ effect, [119]) or dominance [120] interactions. Therefore, we would expect to find benefits to the vocalizer in species that breed colonially, have complex social interactions (i.e. repeated interactions where the previous behaviour affects the current behaviour), repeated territorial interactions and/or dominance hierarchies [17,120].

For the receivers, individual recognition provides two main benefits by first allowing them to identify the signaller and then by allowing them to evaluate the information within the cue or signal based on the signaller's identity. Animals can then use the information to make an informed decision about the present environment and adjust their behaviour accordingly.

At its most basic level, identification of the signaller allows the receiver to determine the potential risks (are they a competitor or do they have higher dominance rank?) and benefits (are they my parents, offspring or possible mate?) of interacting with the vocalizer [17,18]. In species that breed colonially, parents and/or offspring use unique individual calls to identify each other, reducing the likelihood of a mismatch between parents and offspring (thick-billed murre, *Uria lomvia* [63]; macaroni penguin, *Eudyptes chrysolophus* [72]; Australian sea lion, *Neophoca cinerea* [95]).

After determining the identity of the caller, animals may then have the ability to evaluate the information provided within the call. By determining the identity of the caller, the receiver can potentially determine the producer's quality, reliability and the relationship to the receiver. Alarm calls are a common example where IVR allows receivers to evaluate the current call in the context of the signaller's past reliability. In many species, receivers adjust their behaviour in response to alarm calls based on the signaller's reliability, with receivers responding more intensely to those signallers whose calls have been associated with genuine perceived threats (Richardson's ground squirrels, *Spermophilus richardsonii* [35]; yellow-bellied marmots, *Marmota flaviventris* [102]; dwarf mongooses, *Helogale parvula* [103]; Western Australian magpies, *Cracticus tibicen dorsalis* [75]). In some species, alarm calls combine information about the type of predator and the reliability of the signaller, leading to differences in the receiver's behaviour when responding to alarm calls. Both meerkats, *Suricata suricatta*, and vervet monkeys, *Chlorocebus pygerythrus*, alter their responses to alarm calls based on the signaller reliability and the type of predator information within the call (i.e. hawk versus snake calls, meerkats [121], vervet monkeys [122]). By evaluating a signaller's reliability and adjusting their behaviour accordingly, individuals not only reduce their likelihood of expending energy in response to a false threat but also decrease their risk of being eaten by performing the predator-appropriate evasive behaviours.

IVR has the potential to allow individuals to determine the relationship between themselves and the caller and adjust their behaviour based on this relationship. Food-associated calls and territorial calls provide examples where a receiver may evaluate their relationship to the caller and adjust their behaviour accordingly. Ravens, *Corvus corax*, produce ‘Haa’ calls to alert other group members to a particular food source [76]. As these calls contain information about the identity of the caller [76], receivers use them to choose whether to join the caller [76]. Territorial species can also use calls to distinguish their neighbours from strangers. Territorial calls and acoustic recognition of neighbours are widespread throughout the animal kingdom (birds [46]; mammals [84]; frogs [37]; fish [80]). In these species, receivers change their behavioural response based on whether the call they hear is from a neighbour within its own territory [81], a neighbour outside of its territory [123] or a stranger [36]. For social species, vocal communication can also contain information about relatedness [96] and dominance position [98].

## 5. Costs of individual vocal recognition

### (a) Knowing you is exhausting

Recognition systems exist on a continuum, from very general (my species versus all others, male versus female) to the specific (my chick versus other chicks, my mate versus my neighbour), with IVR falling on the specific end. Not all species develop IVR nor would we expect IVR to evolve if less complex recognition will suffice. Species are hypothesized to evolve recognition systems that meet their minimum recognition needs (minimum needs hypothesis) [18,124]. Indeed, we would only expect complex mechanisms for recognition (i.e. IVR systems) to evolve when simpler mechanisms do not provide enough of a recognition advantage owing to the costs of IVR systems [18]. IVR systems can be costly for both receivers and signallers, with both groups expending time and energy in the learning and memory of signals and (for the signaller) production of the signal [125,126]. Complex vocalizations also make signallers an easier target for predators [1]. For the signaller, IVR also provides an additional cost of making it harder to cheat. Recognizable individuals may be less likely to be mistaken for someone else or could be less successful when pretending to be another individual (i.e. offspring being fed by their neighbours mistaking them for their own offspring) [17]. Therefore, we would expect IVR to evolve only when a more general recognition system is not sufficient and when there are enough advantages to being correctly identified to outweigh the costs. Below, we discuss the contexts that favour the evolution of IVR.

## 6. In what contexts is individual vocal recognition often found?

### (a) Are you my mummy?

One of the most well-studied contexts in which vocal recognition can occur is in parent–offspring communication, often tested as IVR-singular. In this context, parents and/or offspring can identify their offspring's/parents' calls from those of others and use them to find their offspring/parents [7,17]. Such studies are typically conducted using playbacks of parent/young and stranger or neighbour calls to determine if the response of the individual is preferential towards their own parent/young. Supporting evidence for such discriminatory ability has been found in both mammals and birds [5,7], though not tested, to our knowledge, in other groups.

For some species, this recognition is a uni-directional relationship, where either the parent can recognize their young, but their young responds similarly to all adult calls (e.g. Mexican free-tailed bats, *Tadarida brasiliensis mexicana* [88]; table 1), or where the young recognize their parents' calls, but their parents do not use their young's vocalizations to find them (e.g. racoons, *Procyon lotor* [6] and tree swallows [65]; table 1). However, although bidirectional relationships do occur, the majority of studies examining parent–offspring IVR focused on either parent or young recognition and the reciprocation is often not adequately tested (table 1). Therefore, in many of the studies showing a uni-directional relationship between parents and offspring, we cannot determine whether the relationship is truly uni-directional or if only one half of the relationship (i.e. parent recognition of offspring or offspring recognition of parents) was tested.

Bi-directional relationships do occur in some species, with both parents and young recognizing each other's calls. Thick-billed murres [63]; reindeer, *Rangifer tarandus* [87]; and domestic sheep, *Ovis aries* [86] all have bi-directional parent–offspring vocal recognition, with both parents and offspring responding preferentially to each other's calls (table 1). Although the mother–offspring relationship is the most commonly reported (likely owing to the majority of mammal species performing maternal-only care), parent–offspring communication includes paternal and non-parental relationships. In razorbills, *Alca torda*, chicks and fathers respond to each other's vocalizations over strangers, but chicks do not respond preferentially to their mother's vocalizations and mothers do not differentiate between calls of their own chicks and those of stranger chicks [62]. In addition, in systems with parent-like individuals (i.e. helpers in cooperative breeding systems), non-parental carers can also be a part of this recognition relationship. In European bee eaters, *Merops apiaster*, a cooperative breeding species, both parents and helpers respond preferentially to begging calls from the chicks in their nest [69].

### (b) Stranger danger

Another well-studied context for IVR is neighbour–stranger recognition. These studies are often testing the ‘dear enemy’ phenomenon—where individuals respond less aggressively to known neighbours than unknown strangers [119], often tested as IVR-singular. Neighbour–stranger recognition is one of the only contexts in which species other than birds and mammals have been tested. Many fish and amphibians are territorial and interact with their neighbours vocally, providing a useful contrast to the results from studies on mammals and birds. In these experiments, a specific playback paradigm or a variant is used to prise apart whether the focal individual recognizes its neighbour from other neighbours and/or strangers. This type of experiment typically involves playing neighbour and stranger calls from different sites on the territory boundary to see how the resident territory holder(s) respond. These playback experiments have found recognition of neighbours by territory holders in a number of species. Bicolour damselfish, *Pomacentrus partitus* [80]; North America bullfrogs, *Rana catesbeian* [21,38]; water rails, *Rallus aquaticus* [57]; ovenbirds, *Seiurus aurocapilla* [50] and black howler monkeys, *Alouatta pigra* [27], to name just a few, all respond more aggressively towards strangers than neighbours on their territory boarder. Alternatively, individuals can also respond more aggressively to neighbours compared to strangers with the same experimental set-up as seen in winter wrens, *Troglodytes troglodytes* [60] and New Zealand bellbirds, *Anthornis melanura* [49]. Although this is a form of individual recognition, questions remain regarding whether the behavioural responses of individuals are more indicative of recognizing a specific individual owing to memory or whether this is simply a habituation response to an individual's vocalizations at a particular location compared to all others [127]. One way to tease apart this issue of habituation versus memory involves altering the neighbour's call so that the change in magnitude either falls *within* the normal within-individual call variation or so that the magnitude of change is *greater* than the normal within-individual variation [127]. Then, by measuring the focal individual's response to the ‘new’ calls—which contain inter-individual variation that is much greater than the focal individual is used to, compared to the calls with normal inter-individual variation—you can try to establish if they respond to the ‘new’ calls as they do to a familiar (habituation) or unfamiliar (memory) individual [127]. Alternatively, researchers could also measure a focal individual's response to their neighbour's call at multiple different sites (i.e. well within the neighbour's territory, at different points along the territory boarder, just inside focal individual's territory) [123]. By incorporating multiple spatial playbacks (as opposed to the standard one site), future studies will be able to distinguish whether species display true IVR or just habituation to an auditory stimulus within a certain space.

### (c) Mate recognition

Another context similar to parent–offspring and neighbour–stranger contexts where individuals identify an individual from all others is mate/partner recognition (IVR-singular). Experiments studying mate/partner recognition also employ playback experiments of mates/partners and either familiar group members or strangers (table 1). This type of recognition has been tested almost exclusively on birds, as mates frequently communicate vocally (announce arrival, negotiate in the nest or duet [123,128,129]) and so has a straightforward testing paradigm. Green-rumped parrotlet, *Forpus passerinus*, females respond more to the contact calls of her mate than other males [44], and kittiwake gulls, *Rissa tridactyla*, respond more to their partner's kittiwake call than either a familiar neighbour's or stranger's call [40]. Often these experiments, like neighbour–stranger experiments, test the mate compared to strangers, but occasionally experiments will also test on a finer scale by including familiar group mates or kin as well (spectacled parrotlet, *Forpus conspicillatus* [78]).

### (d) Social contexts and individual vocal recognition-multiple

Up until now, we have discussed the contexts that are typically studied with an IVR-singular outlook, seeing if individuals can tell one other individual apart from all others. Here, we switch focus to move into contexts that are thought to require IVR-multiple to function (i.e. social or non-categorical contexts). IVR in social or non-categorical contexts (i.e. levels of dominance or degrees of relatedness, etc.) is much less well tested, occurs in multiple contexts, and has only been tested in social birds or mammals.

Many of the circumstances where IVR-multiple is tested involve the natural playback approach, and the results show coarser scale recognition/categorization. For example, many species will differentiate kin from non-kin (Rhesus macaques, *Macaca mulatta* [32]; African elephants [28]; spectacled parrotlets [78]) or familiar individuals versus strangers (pallid bats [107] and ravens [76]).

Fewer studies have successfully shown IVR-multiple, and these studies have almost exclusively used a behavioural psychology or habituation/dishabituation approach to directly ask if a receiver can tell the difference between vocalizations of different individuals. For example, in a habituation/dishabituation task, yellow-bellied marmots, *Marmota flaviventris*, can differentiate between the alarm calls of adult females [130]. During a trained discrimination task, jungle crows, *Corvus macrorhynchos*, could also discriminate between the ‘kaa’ calls of different individuals [30], and in a go no-go experiment, great tits, *Parus major*, discriminated between the songs of different males [29]. IVR-multiple has also been tested using other methods such as manipulation of the relationship between the receiver and the signallers. In Richardson's ground squirrel, *Spermophilus richardsonii*, juveniles respond more to the alarm calls from reliable signallers than unreliable ones [35]. Research on animals in the wild may be able to take advantage of naturally occurring changes in the relationship between individuals (i.e. changes to the dominance hierarchy) to test for IVR-multiple in more species in their natural environment.

### (e) Cross-species individual vocal recognition

IVR can also occur between species, when members of different species regularly interact. When dogs, *Canis familiaris*, were played recordings of their owner's voices paired with images of a stranger (or *vice versa*), they spent longer staring at the mismatched photo than when the voice and photo matched [109]. The authors concluded that not only did dogs know the voice of their owners (IVR) but also ‘actively generate an internal representation of the owner's face when they hear the owner’ [109, p. 17]. Similar results were found in domesticated horses, *Equus ferus*, and rhesus macaques, with both horses and macaques correctly matching the voice of a familiar person with their face (horses [111] and macaques [100]). Subsequent research on dogs found that this phenomenon is not limited to familiar individuals and that dogs can also spontaneously discriminate between unfamiliar speakers (i.e. identifying and distinguishing strangers' voices) [110]. Captive cheetah, *Acinonyx jubatus*, also differentiates between familiar and unfamiliar human voices, responding more frequently, for longer, and more quickly to familiar voices than unfamiliar ones [112]. Heterospecific recognition may occur in the wild as well. For example, semi-free-living red-capped mangabeys, *Cercocebus torquatus*, Campbell's monkeys, *Cercopithecus campbelli* and Guereza colobus monkeys, *Colobus guereza*, which regularly form heterospecific groups, as well as De Brazza monkeys, *Cercopithecus neglectus*, correctly discriminate between contact calls of familiar and unfamiliar DeBrazza monkeys [82]. This last example may, however, not constitute true IVR and instead may be at the level of discriminating between group and non-group members, and all of these results demonstrate that vocal recognition is not limited to members of an individual's species. Indeed, we might expect to find heterospecific IVR in some of the same situations that predict conspecific IVR, namely when individuals have complex social interactions and/or dominance hierarchies (i.e. stable, heterospecific foraging groups). Heterospecific IVR may be one of the most exciting and understudied aspects of IVR research and we encourage future studies to test for IVR in potential heterospecific species (such as mixed foraging groups and mixed defence groups such as cross-species mobbing calls).

## 7. Current gaps and potential solutions for studying individual vocal recognition

Although the field has made huge strides in understanding the drivers behind IVR, how it works and where it occurs, as we have highlighted earlier, there remain some substantial gaps in our knowledge. Here, we identify four categories where we need more research: temporality of recognition, specificity of recognition, taxonomic bias and IVR-multiple testing approaches.

### (a) Temporality of recognition

Very few studies address the temporal scale or specificity of IVR, especially in contexts of IVR-singular, as these behaviours are often seasonally influenced. One way to determine the drivers and underlying mechanisms of IVR is to see whether this behaviour only occurs in a particular season/circumstance (i.e. pre-fledging, while holding a territory, etc.) or it occurs reliably across time. There are a few studies that have looked at the temporal response of individuals to neighbours and strangers across the breeding season [53,60,82]. In all of these studies, individuals only responded differently to neighbours and strangers during the breeding season, suggesting that this recognition may be temporary. If there are no advantages in identifying your neighbours year-round, we may expect the evolution of temporary IVR. For species that only defend territories during the breeding season, for example, IVR may provide only costs outside that season as complex signalling demands energy, takes time and can increase predation risk [4,125]. Therefore, many species may evolve to use temporary IVR only when needed, such as when evaluating members of a lek or in species that form seasonal flocks. Although we may expect temporary recognition to evolve in certain circumstances, temporary recognition could also be a reflection of the playback test. Focal individuals may recognize a neighbour, but there may be no advantage to responding differently until there is a cost to not doing so (i.e. during the breeding season; [4,53,60,125]). By looking within the same season but across years in territorial species with both high and low site fidelity, it may be possible to determine if individuals with high site fidelity respond to last year's neighbours with lower aggression than new, unknown neighbours (i.e. long-term IVR). Experiments could also address kin recognition during and outside breeding seasons to determine if the recognition of vocal signals in colonial breeding species only occurs while young are dependent and in a colonial situation. To test recognition outside of the time when the response to the vocalization itself is beneficial (i.e. breeding season), more robust measures such as habituation/dishabituation or behavioural psychology methods would need to be employed to ensure that a lack of response was a lack of recognition, not a lack of motivation.

In addition to investigating relatively long timescales, studies over shorter timescales may also be highly informative. For example, many social organisms live in relatively fluid populations where groups frequently merge and split (termed ‘fission–fusion’ populations) [131]. Consequently, one may expect that individuals in such populations may either not recognize others at all or may exhibit a dynamical process in which they keep in memory only a relatively small number of individuals at any one time, and that they update this regularly over time. This would give them an effective, cognitively reasonable means of recognizing individuals who are relevant to them throughout their lives.

### (b) Specificity of recognition

Though IVR-singular contexts and approaches (i.e. natural playback) are more straightforward and ecologically relevant to test in the wild, these tests remain inconclusive in regard to the degree or specificity of IVR. Previous studies testing for IVR-singular (i.e. neighbour–stranger, mate recognition, etc.) should be reinvestigated to determine if receivers can recognize multiple individuals, even when multiple individuals do not elicit a unique behavioural response (i.e. when responding to individuals of similar dominance). In particular, all parent–offspring recognition studies should involve testing for bi-directionality, and neighbour–stranger recognition studies should involve all neighbours and maybe even repeated strangers (i.e. slightly more and less familiar strangers).

### (c) Taxonomic bias

Currently, there exists a large taxonomic bias in the literature regarding IVR, with the majority of research focusing on birds and mammals. However, evidence exists in [9], reptiles [13] and fish [11] which suggests that species in these groups are likely to have some form of IVR. To understand the evolutionary and ecological drivers for IVR, we need to sample widely across the animal kingdom and focus not only on species that we predict to have IVR (i.e. social or territorial species) but also species that are not predicted to exhibit IVR based on our hypotheses. Comparisons between related species with and without IVR are a powerful approach in determining the factors favouring the evolution of IVR [17,132]. More studies should investigate IVR-single in territorial fish, reptiles and amphibians and should focus on social fish, reptiles and amphibians to investigate IVR-multiple. Within mammals and birds, a wider variety of species should be examined, focusing on comparative studies of related species that range in sociality to test the role of sociality in IVR-multiple.

### (d) Multi-modality and individual vocal recognition

While many approaches to studying IR, such as IVR, focus on one modality (i.e. vocalizations), IR has been shown to be multi-modal in many cases. Owing to the likely prevalence of multi-modal IR, researchers should be aware of, and take into consideration, other modes of individual recognition, including chemical and visual, that may be combined with vocal signals, especially when studying species such as fish, reptiles, insects and mammals, which are known to use these signals. Many fish use chemical means of communication owing to the properties of water in dispersing chemical cues [133], and these cues can be used for conspecific recognition [133,134]. Multi-modal communication also occurs frequently in mammals, with ring-tailed lemurs (*Lemur catta*) using both olfactory and visual cues to identify conspecifics [135]; domesticated goats (*Capra hircus*) using auditory and visual cues [136] and female Australian sea lions (*Neophoca cinerea*) using olfactory, auditory and visual cues to identify their pups [137]. Therefore, future work on IVR should also consider other forms of communication that individuals may be using.

### (e) Testing for individual vocal recognition-multiple

Few studies have convincingly shown evidence of IVR-multiple despite its likely importance for species with complex social interactions. To better test whether individuals do, in fact, recognize multiple individuals, including over large time scales, more robust approaches need to be taken. Below we outline two such approaches.

#### (i) Behavioural psychology

The behavioural psychology paradigm is the most robust paradigm in many circumstances for testing for IVR-multiple and includes go no-go and discrimination tasks as well as habituation/dishabituation responses. Owing to the difficulty in parsing apart behavioural responses of a focal individual to differences in similar social partners (i.e. multiple individuals treated very similarly by the focal individual across many contexts), directly asking the focal individual if they can tell the difference between the calls of different individuals may be necessary. However, this paradigm relies on the experimenter correctly grouping stimuli and making sure that the stimuli differ only in identity and not some other features (i.e. dominance, sex, age, etc.) that could cause a response without individual recognition. These types of tests also often involve training and take place in the laboratory, limiting our understanding of IVR-multiple in a species' natural environment.

#### (ii) Relationship manipulation

Another way to test for IVR-multiple in its natural ecological context is to change the relationship of the focal individual and the observed individuals. This could occur in a number of circumstances from reliability of a signal (e.g. food or alarm calls) to their relationship to another individual (e.g. dominance contests). By recording an individual's response to a specific individual before and after their relationship is altered, it is possible to determine if the focal individual changes their response based on the changed relationship information (i.e. they must recognize the individual calling to know that the relationship has changed). This paradigm has been used to determine that: 1) individuals attend less to unreliable individuals in anti-predator and foreign troop encounters [35,101,102,137], and 2) females will choose whether or not to engage in extra‐pair matings with a neighbour based on outcomes of playback contests [73]. Males can also alter subsequent singing behaviour in contests based on whether they overheard their current competitor male winning or losing his previous contest [138]. This approach can be very effective, but it is limited to specific types of signals—ones that either (i) encode information about an external state pertinent to all group members (i.e. predator presence, food quality, etc.) or (ii) include information about the individual's current status (i.e. dominance, third-party conflict, etc.) that is pertinent to the focal individual.

## 8. Final thoughts

Individual vocal recognition appears to be a much broader phenomenon than previously believed; however, the explicit study of IVR tends to focus on a small subset of species, completely ignoring entire classes of animals (fish and reptiles). Future research should be spread more evenly across the animal kingdom, including further studies on reptiles, amphibians and fish. In addition, to understand the evolutionary drivers of different types of IVR, more studies need to focus on IVR-multiple and on determining the mechanisms behind IVR-singular (i.e. memory versus habituation). Most importantly, however, our approach and the study of IVR need to be more consistent and methodological. This review has shown the wide variation in how research approaches IVR, and the limitations that often arise owing to ambiguity regarding the level of that IVR individuals may be using or whether they are actively using IVR at all. To effectively study IVR in future, researchers need to first determine what level of IVR they are attempting to examine (IVR-singular or IVR-multiple) and then critically examine their experimental design to ensure that the question they are asking is the question they are intending to answer, removing the ambiguity of habituation and temporal limitations.

## Data accessibility

This article does not contain any additional data.
